# Comparison of Glyphosate Detection by Surface-Enhanced Raman Spectroscopy Using Gold and Silver Nanoparticles at Different Laser Excitations

**DOI:** 10.3390/molecules27185767

**Published:** 2022-09-06

**Authors:** Lara Mikac, István Rigó, Marko Škrabić, Mile Ivanda, Miklós Veres

**Affiliations:** 1 Molecular Physics and New Materials Synthesis Laboratory, Ruđer Bošković Institute, Bijenička 54, 10000 Zagreb, Croatia; 2 Department of Applied and Nonlinear Optics, Institute for Solid State Physics and Optics, Wigner Research Centre for Physics, 1121 Budapest, Hungary; 3 Department of Physics and Biophysics, School of Medicine, University of Zagreb, Šalata 3b, 10000 Zagreb, Croatia

**Keywords:** SERS, glyphosate, substrate, pesticides, silver, gold, colloid

## Abstract

Glyphosate is one of the most widely used pesticides in the world, but it has been shown to persist in the environment and therefore needs to be detected in food. In this work, the detection of glyphosate by surface-enhanced Raman scattering (SERS) using gold and silver nanoparticles and three different commonly used laser excitations (532, 632, and 785 nm wavelengths) of a Raman microscope complemented with a portable Raman spectrometer with 785 nm excitation is compared. The silver and gold nanosphere SERS substrates were prepared by chemical synthesis. In addition, colorimetric detection of glyphosate using cysteamine-modified gold and silver nanoparticles was also tested. The best results were obtained with Ag NPs at 532 nm excitation with a detection limit of 1 mM and with Au nanoparticles at 785 nm excitation with a detection limit of 100 µM. The SERS spectra of glyphosate with cysteamine-modified silver NPs improved the detection limits by two orders of magnitude for 532 nm excitation, i.e., up to 10 µM, and by one order of magnitude for 632 and 785 nm excitation wavelengths.

## 1. Introduction

Pesticides are chemical or microbiological agents that control, destroy, repel, or mitigate the effects of various pests such as insects, rodents, and weeds. Unfortunately, pesticide use disrupts the natural balance in the environment, leads to resistance, and can cause water and food contamination. Their use in crop protection can also result in the absorption of these substances by plants, which are later used in food [1,2]. However, even the best regulations cannot fully protect consumers from excessive use of additives. Controlling the production and use of pesticides and analyzing their presence in food, water, and the environment are expensive and complex [3]. Gas and liquid chromatography combined with UV spectroscopy, nuclear magnetic resonance or mass spectroscopy are the most common analytical methods used in food analysis [4,5,6,7]. These techniques are accurate and sensitive but have certain limitations: they are complex and require trained personnel, they are time-consuming, and the analysis must be performed in a laboratory. Therefore, it is of great interest to develop simple, rapid, inexpensive, and sensitive methods for the detection of pesticides in samples with low concentrations.

Raman spectroscopy is fast and simple, requires no or minimal sample preparation, and its advantage is the possibility of analysis in aqueous solutions. However, problems such as insufficient sensitivity and the frequent occurrence of fluorescence limit the capabilities of Raman spectroscopy. Since its discovery, surface-enhanced Raman scattering (SERS) has gained great popularity as a sensitive method for studying the vibrational properties of molecules adsorbed on rough metal surfaces of nanometer size, even at low concentrations [8,9]. It is widely accepted that two mechanisms are responsible for enhancing the intensity of the Raman signal during SERS: electromagnetic (EM) and chemical effects. The electromagnetic enhancement is a consequence of the interaction of the external electromagnetic fields of the incident and/or scattered light with those generated by the plasmon excitation of the nanoscopic metal substrate, and the chemical enhancement, in which the adsorbed analyte molecule transfers electrons to the metal substrate, causing the formation of chemical bonds between the metal surface and the analyte molecule [10].

The choice of excitation wavelength depends entirely on the material under investigation. Since the Raman shift is small compared to the bandwidth of the local surface plasmon resonance (LSPR), both the incident and scattered light fields can be amplified. Therefore, the electromagnetic enhancement factor is approximately equal to the field enhancement to the power of four. By tuning the excitation laser to the surface plasmon resonance, it is possible to maximize the local electromagnetic field enhancement and obtain the largest signal [11]. The LSPR wavelength of a plasmonic material, in our case Au and Ag NPs, depends on their size and shape and ranges from 500 to 800 nm for gold and 400 to 700 nm for silver. Moreover, the plasmonic peak shows a red shift with increasing nanoparticle size or with the formation of NP aggregates. Therefore, the wavelength of the excitation source must be close to the plasmonic resonance peak of the plasmonic material. According to the literature, the best excitation is a little blue-shifted with respect to the LSPR maximum of the SERS substrate [12].

Another important point is the choice of an excitation wavelength that does not produce photoluminescence, reduces the noise level and in this way gives a Raman spectrum with a better signal-to-noise ratio. It is well known that the strength of Raman scattering is proportional to the fourth power of the excitation frequency, i.e. inversely proportional to the fourth power of the wavelength. Therefore, it is reasonable to expect a higher Raman signal from a given sample when shorter excitation wavelengths are used.

One of the main advantages of the SERS method is the possibility to analyze photoluminescent compounds, since the latter is quenched near the SERS substrate where the plasmonic enhancement of the Raman signal is the highest [13]. The analysis of many compounds is performed in situ using small portable Raman spectrometers [14,15]. The sensitivity of the SERS method depends on the nature of the substrate, which can be either a colloidal solution of metal nanoparticles or a metal surface with a suitable nanostructured topology [16,17]. The SERS method usually provides signal amplification of the order of 10^6^, but in extreme cases amplifications of ~10^14^–10^15^ are possible, which is sufficient for the detection of one or a few molecules [18]. Today, suspensions of silver and gold nanoparticles (colloids) are most commonly used as SERS substrates [19].

The sensitivity and repeatability of the Raman signal can be significantly increased by using metal particles of precisely defined size and shape [20]. The particle size suitable for SERS ranges from 10 to 80 nm. The size and shape of the particles can be controlled in part by the choice of colloid preparation method. Pesticides, antibiotics, drugs, melamine, illegal food dyes, and toxic proteins, as well as other contaminants such as perchlorate and polycyclic aromatic hydrocarbons, have been studied in detail using the SERS method [21,22,23,24,25,26,27].

Glyphosate (*N*-(phosphonomethyl)glycine) (Table 1) is the most widely used active ingredient for crop protection worldwide [28]. Some of the commercially available products are Roundup biactive (manufactured by Monsanto), Total 480 SL (manufactured by Nufram, Genera) and Hercules super (manufactured by Cheminova). When glyphosate is applied directly before harvest, it can be found in food. Studies on the harmfulness of glyphosate are still ongoing [29,30]. Glyphosate inhibits amino acid synthesis and acts as an endocrine disruptor, affecting hormone secretion and fertility [31,32]. Studies show that glyphosate stimulates the growth of breast cancer cells in women via estrogen receptors [33].

The detection of glyphosate is mainly based on chromatographic methods. In gas chromatography, glyphosate is derivatized to a volatile and thermally stable derivative [34,35]. In liquid chromatography, glyphosate derivatives are detected with a UV–visible detector and a fluorescence detector [36]. Ion chromatography and capillary electrophoresis are also used for analysis [37,38].

Costa and colleagues published a paper in 2012 that investigated the binding of organophosphorus pesticides (including glyphosate) using SERS spectroscopy [39]. The results indicated that glyphosate binds to silver nanocubes through the phosphate group.

Xu and co-workers proposed a simple and sensitive method for the determination of glyphosate by combining the ninhydrin reaction and SERS [40]. They found that the product of the ninhydrin reaction was SERS active and directly correlated with glyphosate concentration. The detection limit of the proposed method for glyphosate was 1.43 × 10^−8^ mol dm^−3^. A colorimetric method for in situ detection of glyphosate on plant tissues using cysteamine-modified gold nanoparticles was also developed [41].

In this study, glyphosate was detected directly using SERS. SERS substrates based on colloidal gold and silver solutions were prepared by chemical synthesis. Three laser excitation sources with wavelengths of 532, 632, and 785 nm were used for SERS measurements. The effect of excitation wavelength on SERS enhancement of glyphosate using gold or silver substrates was investigated. The choice of SERS substrate and laser excitation with the best signal enhancement was evaluated by comparing glyphosate signal intensities. Colorimetric detection of glyphosate was also performed using Au and Ag nanoparticles modified with cysteamine. Samples suitable for colorimetric detection were also tested with SERS.

## 2. Results and Discussion

### 2.1. Characterization of Nanoparticles

**Figure 1:**
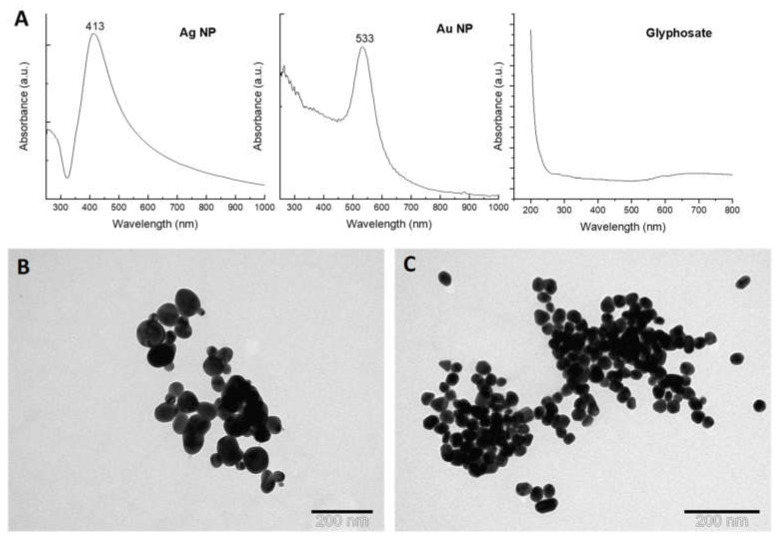
(**A**) UV–Vis spectra of synthesized nanoparticles and glyphosate solution (2.4 mM); TEM images of (**B**) Ag NPs and (**C**) Au NPs.

Ag and Au nanoparticles were synthesized for use in the SERS measurements. The characteristics of the samples are listed in Table 2. The obtained colloidal solutions were characterized by UV–Vis absorption spectroscopy and transmission electron microscopy. Figure 1A shows the UV–Vis absorption spectra of the synthesized Ag NPs with a well-defined LSPR band at 413 nm and Au NPs with a plasmon band at 533 nm. These values are expected for spherical Ag and Au NPs in the size range of 20 to 100 nm. The relatively narrow width of the plasmon peaks indicates the formation of uniform sized NPs in the colloids. The UV–Vis absorption spectrum of glyphosate solution is also shown in Figure 1A. It can be seen that this analyte does not contain intense absorption bands in the visible region.

TEM measurements were performed to observe the size and morphology of the synthesized NPs (Figure 1B,C). TEM images show spherical NPs in both samples. The Au colloid consists of particles with only one size, while two particle sizes are present in the Ag colloid. The calculated mean particle size for the Au colloid is 27.3 nm and for the Ag colloid is about 32 and 72 nm, respectively. The hydrodynamic size distributions by volume and hydrodynamic diameters for the colloidal samples are listed in Table 2. The results of the DLS measurements indicate that the hydrodynamic diameters of the NPs agree well with the sizes estimated using TEM. Measurements of the zeta potential (ζ) indicate negatively charged stable particles that are not prone to spontaneous aggregation. The pH of the Ag and Au colloidal suspensions was found to be 6.5 and 4.7, respectively.

### 2.2. Raman and SERS Measurements

**Figure 2:**
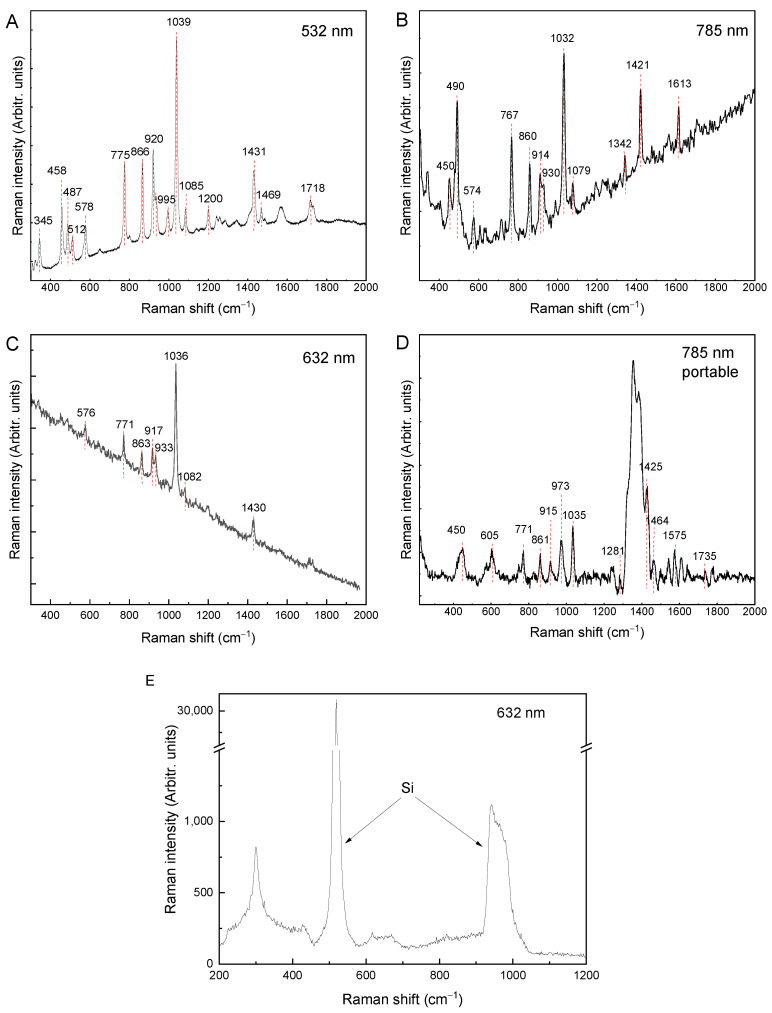
Raman spectra of glyphosate powder recorded with different laser excitations: (**A**) 532 nm (**B**) 785 nm, (**C**) 632 nm, (**D**) 785 nm (portable Raman; baseline corrected), and (**E**) Raman spectrum of 10 mM glyphosate solution excited at 632 nm on Si wafer.

The Raman spectra of the glyphosate powder sample were recorded with 532, 632, and 785 nm laser excitations and are shown in Figure 2. Fairly good glyphosate Raman bands are observed in all spectra. Photoluminescence is present but does not prevent detection of the Raman peaks at any of the excitation wavelengths. The spectral profile depends strongly on the excitation wavelength, although the most prominent Raman band at ≈1032 cm^−1^ is present in all spectra, along with peaks at about 450, 574, 767, 860, 914, and 1421 cm^−1^ (the peak positions show some variance depending on the excitation wavelength). The peak positions of glyphosate from the literature and those observed in our experiment are listed in Table 3.

In addition to the difference in the photoluminescence background, the main difference between the spectra is the change in the intensity ratio of the bands. For example, at 632 nm excitation, the band at 1036 cm^−1^ (or the corresponding band at 1039 cm^−1^/1032 cm^−1^) has a higher intensity under all three laser excitation energies, and its intensity is several times higher than that of the other bands. At 532 nm excitation, the bands in the 700–950 cm^−1^ range have a higher intensity than the peaks in the 400–600 cm^−1^ region, in contrast to 785 nm excitation, where the band at 490 cm^−1^ has a higher intensity compared to the bands between 700–950 cm^−1^. With the portable Raman system (Figure 2D), the resolution and intensities are somewhat weaker relative to the noise. In addition, impurities or effects of laser heating on the sample can be seen in the 1300–1400 cm^−1^ region, where a broad band appears, presumably because of the deterioration and amorphization of the glyphosate by the laser radiation. Figure 2E shows the Raman spectrum of the glyphosate solution as well. No glyphosate Raman bands are present; only the peaks originating from the Si wafer used as a solid substrate can be observed.

The SERS substrates used to detect glyphosate were silver and gold NPs, and the same excitation wavelengths were used. When gold NPs were used as SERS substrate, no signal appeared at 532 nm excitation. When silver NPs were used together with the 785 nm excitations, the SERS signal was low; thus, these were not included in the study. This can be explained by the fact that gold colloids have a plasmon maximum at about 520 nm, but after the formation of aggregates of Au nanoparticles, the plasmon resonance shifts toward longer wavelengths, making the use of 785 nm excitations more favorable for gold NPs.

**Figure 3:**
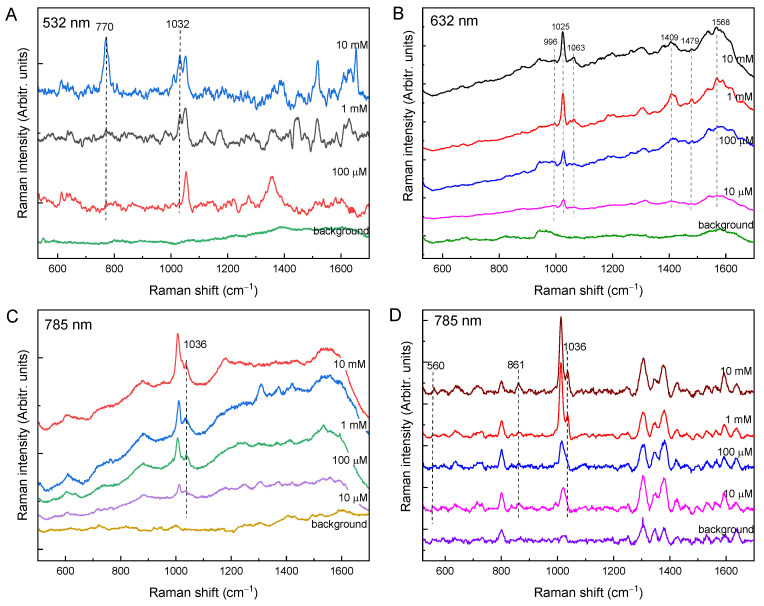
SERS spectra of different glyphosate concentrations: (**A**) with Ag NPs at 532 nm excitation; (**B**) with Au NPs at 632 nm excitation; (**C**) with Au NPs at 785 nm excitation; (**D**) with Au NPs at 785 nm excitation (baseline corrected; portable Raman).

As previously reported in the literature, not all Raman bands of glyphosate appear in the SERS spectra [38]. For example, when using a 532 nm excitation with Ag NPs, two prominent SERS glyphosate peaks are located at 1032 and 770 cm^−1^ (Figure 3A). Using Au NP and 632 nm excitation, the most prominent peak is at 1025 cm^−1^. Peaks at 996 and 1063 cm^−1^ also occur, but with lower intensity, along with several peaks in the 1400–1600 cm^−1^ region. A colloidal Au solution and excitation at 785 nm resulted in spectra in which the peaks at 1010 and 1036 cm^−1^ are most pronounced. Using a portable Raman instrument and gold NPs, the most intense peaks are found at 1010 and 1036 cm^−1^, and peaks at 560 and 861 cm^−1^ are also present. The band at about 1010 cm^−1^ is more intense than the SERS glyphosate band at 1036 cm^−1^, and its intensity decreases with decreasing concentration, but there was no evidence in the literature that this band was arising from glyphosate. In our opinion, this band could correspond to the 996 cm^−1^ peak, which was shifted to higher frequencies due to the interaction of glyphosate with the gold nanoparticles. The absorption of the glyphosate molecule on the Ag/Au NPs is most likely through the P atom, as indicated by the vibrational enhancement of the band at 1036 cm^−1^ that occurs in all spectra. All observed Raman and SERS bands are listed in Table 3 along with the bands and tentative assignments from the literature.

Considering the detection limit under the given conditions, the best results (detection limits) were obtained with 532 nm excitation for silver nanoparticles of 1 mM and 785 nm excitation for gold NPs up to a concentration of 100 µM. Although these detected glyphosate concentrations are high compared to the literature data, the aim of this work was not to obtain low detection limits, but to compare the SERS measurement conditions for different NPs and excitation wavelengths. For example, De Goes et al. synthesized citrate-stabilized silver nanoparticles prepared by pulsed laser ablation in liquids for the detection of glyphosate in water [42]. The detection limit for glyphosate in water obtained with SERS was 1.3 mg L^−1^. Feis and co-workers concluded that the relevant differences in intensities and wavenumbers between SERS and Raman spectra of glyphosate must be due to the effects caused by adsorption on the nanoparticles [43]. Recently, an indirect SERS sensing assay was developed for the determination of glyphosate (Gly) in tap water [44]. The mechanism of detection was based on relieving the inhibitory effect of L-cysteine (L-cys) on an Au-Pt nanozyme by the association of Gly with L-cys by divalent copper ions (Cu^2+^). The detection limit and quantification limit of Gly were reported to be 5 and 10 μg L^−1^, respectively.

**Table 3:** Experimental glyphosate peak positions (cm^−1^) observed in Raman and SERS spectra.

Raman [43]	SERS [43]	Raman 532 nm	SERS 532 nm	Raman 632 nm	SERS 632 nm	Raman 785 nm (Portable)	SERS 785 nm (Portable)	Raman 785 nm	SERS 785 nm	Tentative Assignment from [39,45]
		345		339				339		
455		458		454		450		452		δ(PO_3_) + δ(NCCO) or ρ(CH_2_) + δ(OH)
485		487		484				490		δ(HOPO) + ρ(PCN)+δ(NCC) + δ(HOCO)+ρ(CH_2_) or δ(OH) + ρ(CH_2_) + (PO_2_)
509		512						508		δ(HOPO) + δ(CNC)+δ(HOCO) + ρ(CH_2_) or δ(OH) + δ(CH)
576	565 (broad)	578		576		573	560	574		δρ(PO_3_) + skel(NCCOO) or δ(OH) + δ(HO-C=O)
						605		606		
646		650								ν(PC) + δ(NCC) + δ(COO)
	720									ν(PC) or δ(NH) + ρ(CH_2_) + ν(P-OH)
773	770	775	770	771		771		767		ν(PC) or δ(NH) + ρ(CH_2_) + ν(P-OH)
798	799	801								ν(PC) + ρ(CH_2_) + ρ(NH_2_) + ν(CCOO)
	832									ν(P-OH)
	889									ρ(CH_2_)
864		866		863		861	861	860		ν(C-C) or ρ(CH_2_) + δ(NH) + ν(C-C)
917		920		917		915		914		CNCC skel.
933	936	935		933				930		ν_s_(PO_3_) + ν(PC)
979 [46]						973				ρ(C2H_2_)
992	974	995			996		1010	988	1010	ν_s_(PO_3_) + τ(CH_2_) + ρ(NH_2_) + CNCC skel. or ρ(CH_2_) + δ(OH)
1036	1023	1039	1032	1036	1025	1035	1036	1032	1036	ν(C-N)/CNCC skel. + ν_a_ (HOPO_2_)/ν_a_(POO) or ν_s_(PO_2_) + δ(OH)
1081	1051	1085		1081	1063			1079		ν_a_(PO_3_) + ν(C-N) or ν(C-N) + ν(C-OH)
1136		1140		1136						ν_a_(POH)
1160		1161		1158						δ(CH_2_+NH_2_ + CH_2_) + ν(COH)
1196	1189	1200		1198	1185			1193		δ(CH_2_+NH_2_ + CH_2_) + ν(COH) + ν(CN) or τ(CH_2_) + δ(OH)
1238		1242		1241						ν(POH) + τ(CH_2_) + ν(COH) + δ(CNC) or τ(CH_2_)
1255	1255	1259		1255						ν(PC) + ν(POH) + ω(CH_2_) or ω(CH_2_)
1281		1285				1281				ω(CH_2_) + ωτ(CH_2_) + δ(COH) + ν(PC)
1340	1313	1343						1342		ωτ(CH_2_) + δ(COH) + δ (CNC)/ν(POH) + … or ω(CH_2_) + ν(C-C)
1400		1407 (shoulder)		1425	1409					δ(CH_2_) + ν(CCOH)
1427	1435	1431		1430		1425		1421		δ[C(2)H2] + δ(POH) or δ(CH_2_)
1431 (shoulder)	1397	1436 (shoulder)								δ[C(4)H2] + δ(POH) or δ(CH_2_)
1466		1469		1464	1479	1464				ν(CC) + δ(CNH)/τ(NH_2_)
1483		1487								ω(NH_2_) + δ(POH)
1566		1569			1568	1575				δ(NH_2_)
						1610		1613		
1714		1717		1710						ν(C=O)
1728		1732		1727		1735				ν(C=O)

s: symmetric; as: asymmetric; ν: stretching; δ: bending; ρ: rocking; ω: wagging; τ for twisting; skel: skeleton vibration.

### 2.3. Colorimetric Assay

Further experiments were performed to investigate the possibility of colorimetric detection and to determine whether better SERS signals of glyphosate are obtained when cysteamine is used as the molecule through which it binds to Au or Ag NPs. In addition, visual detection based on the color change of NPs is interesting because of its simplicity.

**Figure 4:**
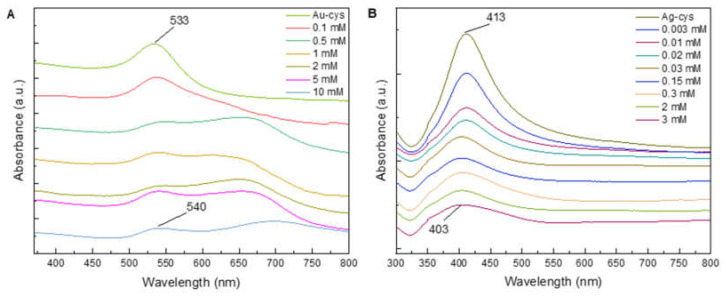
UV–Vis absorption spectra after the addition of different concentration of glyphosate for: (**A**) Au NPs and (**B**) Ag NPs.

Colorimetric detection based on the color change of colloidal particle solutions is often used as a simple analytical method because the addition of analytes leads to the aggregation of NPs, which changes the localized surface plasmon resonance (LSPR) wavelength. The higher the analyte concentration, the stronger the aggregation of NPs due to the stronger interaction between the analyte and NPs modified with cysteamine. Here, the aqueous solution of glyphosate was added to a previously prepared aqueous dispersion of cysteamine-coated gold and silver NPs, resulting in the alteration of the surface properties of the NPs and thus a change in the color of the dispersion. The thiol group of the cysteamine adheres to the surface of the NPs by forming a bond with the S atom, leaving the amino group free for interactions. Furthermore, the glyphosate molecule (Table 1) contains carboxyl (-COOH) and phosphonyl (-PO_3_H_2_) in its structure and shows strong affinity for the amino group. The adsorption of glyphosate on Au NPs coated with cysteamine leads to crosslinking between the particles and thus to a red shift in the absorption spectrum [47]. Citrate-coated NPs do not change color after the addition of glyphosate, as reported in the literature [41,47]. For glyphosate detection, cysteamine-modified NPs were mixed with different concentrations of glyphosate, and a red to purple color change was observed for gold NPs and a yellowish to brownish color shift for silver NPs. The UV–Vis absorption spectra of Au and Ag NPs modified with cysteamine and mixed with different concentrations of glyphosate are shown in Figure 4. It can be seen that when Au NPs were used, the SPR band shifted to higher wavelengths with increasing glyphosate concentration, and a new additional absorption band appeared in the long-wavelength region. It was also found that when glyphosate was added to Ag NPs modified with cysteamine, a blue shift of the LSPR peak occurred. As the concentration of glyphosate increased, the intensity of the surface plasmon resonance band decreased. At the same time, a broadening of the peak was observed with a shift of its maximum to the region of shorter wavelengths, but no additional absorption bands appeared. This behavior is unusual since the LSPR peak was expected to shift to higher wavelengths due to the formation of Ag aggregates. According to Sahu et al. the blue shift of the LSPR peak could be explained by the interaction between the collective longitudinal oscillation of the electrons and the surrounding medium [48]. Several studies have shown that surfactants can reorganize depending on the thermodynamic environment in which they are located [49].

#### SERS Detection of Glyphosate in Colorimetric Assay

**Figure 5:**
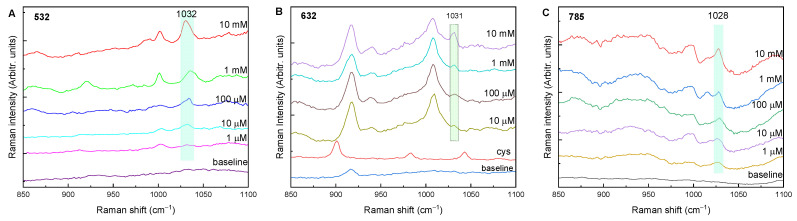
SERS spectra of different glyphosate concentrations with cysteamine-modified NPs: (**A**) Ag with 532 nm excitation, (**B**) Au with 632 excitation and (**C**) Au with 785 nm excitation. (cys: cysteamine powder).

After the colorimetric measurements, the SERS signal of different glyphosate concentrations was determined for gold or silver NPs modified with cysteamine. Figure 5 shows the SERS spectra of the corresponding glyphosate concentrations for 532, 632 and 785 nm laser excitations. As the analyte concentration decreases, the intensity of the glyphosate band at about 1030 cm^−1^ also decreases. Some other bands are also present in the spectra (e.g., the band at 917 cm^−1^ when excited with 632 nm, or at 1002 cm^−1^ when excited at with 532 nm), but their intensity does not change with concentration, and they cannot be said to originate from glyphosate (or cysteamine).

Using NPs modified with cysteamine, good glyphosate bands at ≈1030 cm^−1^ were obtained with Ag NPs and excitation at 532 nm and with Au NPs and excitations at 632 and 785 nm. This peak is present in all spectra, and its intensity decreases with decreasing concentration. Therefore, it can be used as a glyphosate indicator, and according to the SERS data, the cysteamine-modified colorimetric assay increases the detection limit to 10 µM in all three cases.

## 3. Materials and Methods

### 3.1. Materials

Gold(III)chloride trihydrate, trisodium citrate dihydrate, hydroxylamine hydrochloride, glyphosate (*N*-(phosphonomethyl)glycine) powder, sodium nitrate, and cysteamine hydrochloride (≥98%) were provided by Merck. Silver nitrate was provided by Kemika (Zagreb, Croatia). All chemicals were of analytical grade and were used without further processing. High purity water with a resistivity of 18 MΩ cm^−1^ was used for all experiments.

### 3.2. Synthesis of Nanoparticles

#### 3.2.1. Synthesis of Silver Nanoparticles (Ag NPs)

The colloidal suspension of Ag NPs was synthesized by a modified Leopold, Lendl method [50]. A total of 10 mL of AgNO_3_ (10 mM) was added to 90 mL of a hydroxylamine hydrochloride solution (1.67 mM) containing 3.33 mM sodium hydroxide at room temperature with stirring. The mixture was stirred for 15 min.

#### 3.2.2. Synthesis of Gold Nanoparticles (Au NPs)

The colloidal suspension of Au NPs was synthesized by a modified Turkevich method using sodium citrate as reducing agent and a loosely bound capping agent [51]. According to the procedure, 200 mL of HAuCl_4_ (1 mM) was vigorously stirred and heated in a round bottom flask with reflux condenser. Then, 10 mL of 38.8 mM sodium citrate was rapidly added to the boiling solution and boiled for 15 min until the color changed from pale yellow to wine red. The resulting 0.95 mM colloid solution was stable at 4 °C for several months.

#### 3.2.3. Modification of NPs with Cysteamine

Cysteamine-modified NPs were prepared according to the protocol described in the literature [41,52]. First, 50 μM cysteamine solution was added to the NPs solution in a volume ratio of 1:100. The mixed solution was stirred for 2 h and then washed to remove the excess cysteamine. The final cysteamine–NP product was redispersed in water and stored at 4 °C. Next, 0.2 mL of 1 mM acetate buffer and 0.2 mL of cysteamine-NP were mixed and incubated for 5 min at room temperature. Then, 0.2 mL of glyphosate solution at different concentrations was added to the mixture and equilibrated for 10 min. UV–Vis absorption spectra were recorded in the wavelength range of 350–800 nm.

### 3.3. SERS Sample Preparation

The stock solution of glyphosate was prepared in water. The colloidal suspension, the solution of the inorganic salt, and the various dilutions of glyphosate prepared previously were mixed in appropriate proportions. The glyphosate water solutions used for SERS experiments were 10 mM, 1 mM, 100 µM, 10 µM and 1 µM. In previous experiments, it was found that the best enhancement of the signal was obtained with the following ratios of nanospheres: 80 μL nanospheres suspension, 10 μL pesticide sample and 10 μL aggregating agent (1 M inorganic salt). SERS experiments were performed in two ways. In the first method, the obtained colloidal suspension, aggregating agent (salt), and analyte molecule at the appropriate molar concentration were vortexed for 10 s and then placed in a glass tube, and the Raman signal was measured. In the second method, a drop (2 μL) of the mixture was dropped onto the clean substrate and dried. The substrate was positioned under the microscope of the Raman instrument.

### 3.4. Nanoparticles Characterization

The size and size distribution of nanoparticles were studied using a UV–Vis spectrometer (Cary, Agilent, Santa Clara, CA, USA) and a transmission electron microscope (JEM 1010, Jeol, Tokyo, Japan). For TEM measurements, the particles were dropped onto a copper grid coated with Formvar and allowed to dry. ImageJ software was used to determine the particle size distributions.

Dynamic light scattering (DLS) was used to further evaluate the diameter distribution. DLS measurements were performed using the Zetasizer Nano S, and zeta potential measurements were performed using the Zetasizer Nano Z (Malvern, Malvern, UK). Size distributions were reported as distributions by volume, and results are presented as the mean of at least 3 measurements. Zeta potential was reported as the mean of three measurements. The pH measurements were performed using a pH meter calibrated with 4.005 and 7.00 buffer solutions. All measurements were performed at room temperature.

Raman spectra were recorded using an inVia Raman spectrometer (Renishaw, Wotton-under-Edge, UK) and the Cora 5001 (Anton Paar, Graz, Austria) fiber portable Raman spectrometer. The Renishaw inVia micro-Raman spectrometer was connected to a Leica microscope, and lasers with wavelengths of 532, 632, and 785 nm were used as excitation sources, with the laser beam focused into a spot of 1–2 μm diameter on the sample. For measurements at an excitation wavelength of 785 nm, the Cora 5001 was equipped with a CCD detector cooled to subambient temperature and a diode laser with power up to 450 mW. The spectral resolution was 6 to 9 cm^−1^. The measurement times and laser intensities are given in Table 1. To reduce photodegradation of the samples, the laser excitation power was kept low for all excitations. The Raman spectra of the samples were recorded with a ×50 microscope objective, and the experiments were performed at room temperature. The range studied was between 300 and 2000 cm^−1^, and the Raman band of a silicon wafer at 520 cm^−1^ was used to calibrate the spectrometers. Raman measurements were made at several locations on the substrate surface, and the measurement was repeated at the location where the SERS enhancement was best.

## 4. Conclusions

In this work, a comparison of glyphosate detection with Raman spectroscopy was performed using three different excitation wavelengths (532, 632, and 785 nm). The obtained Raman bands of glyphosate powder were compared with those from the literature. The characteristic Raman glyphosate peak at ≈1030 cm^−1^ appeared in all spectra and was used for glyphosate detection in further work. Most Raman bands were detected with a 532 nm excitation wavelength.

Au and Ag nanoparticles were synthesized and used as SERS substrates. The SERS spectra of different glyphosate concentrations were recorded with Ag nanoparticles at 532 nm excitation and with Au nanoparticles at 632 and 785 nm excitation. The most prominent glyphosate band was found at ≈1036 cm^−1^. All SERS spectra showed reasonable enhancement of the Raman signal when used for the respective excitation wavelength. The best results were obtained with Ag nanoparticles at 532 nm excitation with a detection limit of 1 mM and with Au nanoparticles at 785 nm excitation with a detection limit of 100 µM.

For colorimetric detection of glyphosate, cysteamine-modified NPs were mixed with different concentrations of glyphosate, and change in the color was observed. When cysteamine-modified Au NPs were used, the LSPR band shifted to higher wavelengths with increasing glyphosate concentration, but when glyphosate was added to modified Ag NPs, the position of the LSPR peak unexpectedly shifted to the blue spectral region. The SERS spectra of glyphosate with cysteamine-modified silver NPs improved the detection limit by two orders of magnitude for 532 nm excitation, i.e., up to 10 µM, and by one order of magnitude for 632 and 785 nm excitation. The broader glyphosate band at ≈1030 cm^−1^ was observed and used to achieve the detection limit of 10 µM for all three excitation wavelengths.

## Figures and Tables

**Table 1:** SERS measurement parameters for glyphosate.

Molecular Structure	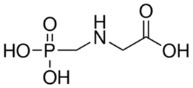			
Excitation Wavelength (nm)	532	632	785	785 (portable)
Laser Intensity	0.05%, 32 µW	1%, 90 µW	10%, 3.5 mW	50 mW
Measurement Time	30 s	60 s	10 s	10 s

**Table 2:** The characteristics of the Ag and Au samples.

Sample	LSPR λmax (nm)	Zeta Potential (ζ) (mV)	Hydrodynamic Diameter ^1^ (nm)	pH	DTEM (nm)
Ag NPs	413	−33.7	17.4 (98%), 120.8 (2%)	6.5	32.1 ± 6.7; 71.9 ± 13.8;
Au NPs	533	−12.9	27.6 (93%), 131.5 (6%)	4.7	27.3 ± 4.3

^1^ Percentages in parentheses indicate ratios based on volume distributions.

## Data Availability

The data presented in this study are available on request from the corresponding author.

## Sample Availability

Samples of the silver and gold colloids are available from the authors.
